# Supplementary figures and images for: A novel household water insecurity scale: Procedures and psychometric analysis among postpartum women in western Kenya
Source: PLoS One. 2018 Jun 8;13(6):e0198591. doi: 10.1371/journal.pone.0198591 (PMC5993289; doi:10.1371/journal.pone.0198591)

**S1 Fig. Integration of Delphi Method with Focus Group Discussions**

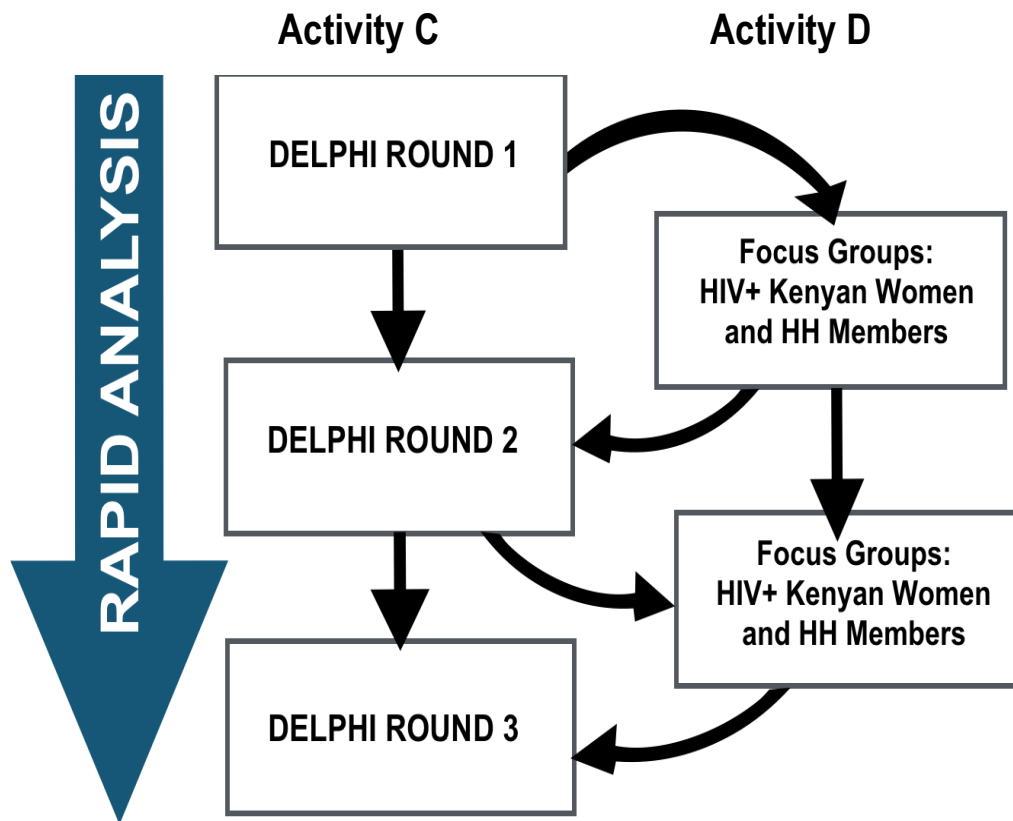

Supplement: S1 Fig — (PDF) [file pone.0198591.s001.pdf]

**S3 Fig. Scree plot showing cut-off point for retained scale factors using parallel analysis**

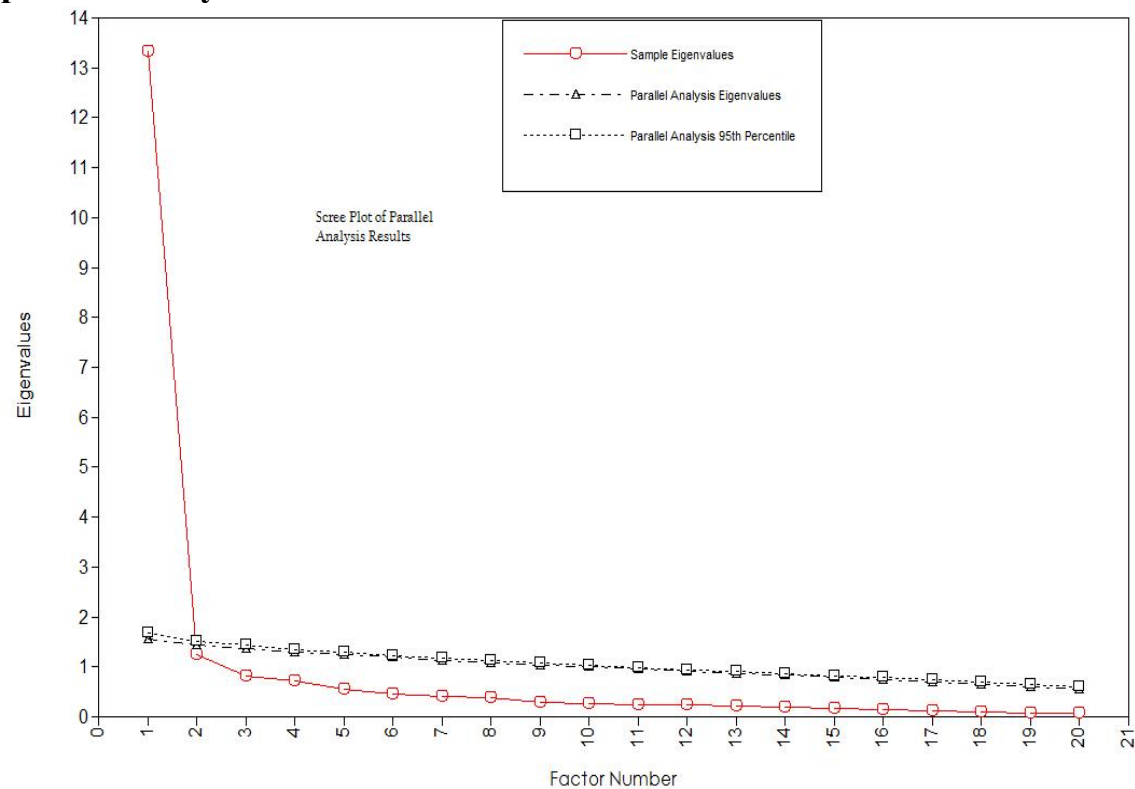

Supplement: S3 Fig — (PDF) [file pone.0198591.s003.pdf]
