# Supplementary material for: A novel household water insecurity scale: Procedures and psychometric analysis among postpartum women in western Kenya
Source: PLoS One. 2018 Jun 8;13(6):e0198591. doi: 10.1371/journal.pone.0198591 (PMC5993289; doi:10.1371/journal.pone.0198591)

S2 Fig. Scree plot showing cut-off point for retained scale factors using exploratory factor analysis

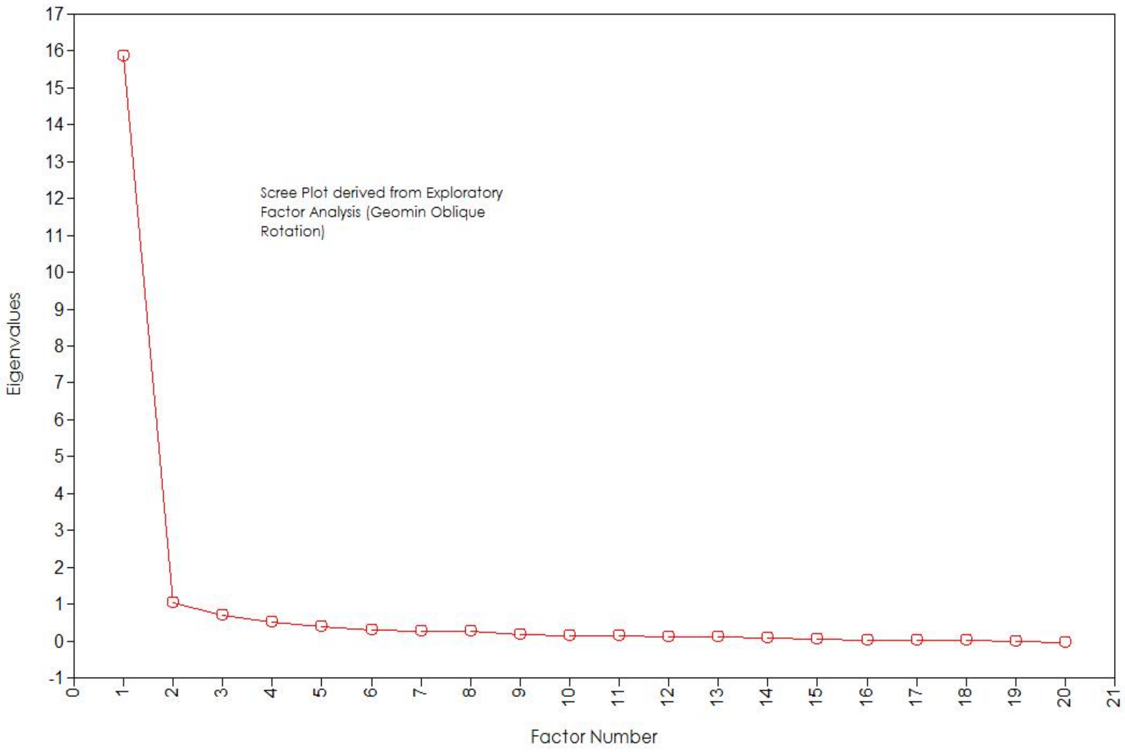

Supplement: S2 Fig — (PDF) [file pone.0198591.s002.pdf]
