# Supplementary material for: A novel household water insecurity scale: Procedures and psychometric analysis among postpartum women in western Kenya
Source: PLoS One. 2018 Jun 8;13(6):e0198591. doi: 10.1371/journal.pone.0198591 (PMC5993289; doi:10.1371/journal.pone.0198591)
Supplement: S1 Table — (PDF) [file pone.0198591.s004.pdf]

S1 Table. Delphi questions on household water insecurity

**Delphi Round 1 questions**
Q1. When you read or hear the phrase "household water security" what do you think of?<br>Q2. How would you define household water insecurity?<br>Q3. Think of low and middle income countries with which you are familiar. What are the 4 or 5 most important household activities for which water is needed? For each one, why do you consider these activities to be the most important?<br>Q4. Think again of a low- and middle-income country. What do you think are some consequences that would be associated with insufficient water in the household?<br>Q5. Think again of a low- and/or middle-income country. What are some of the first household activities that would be impacted by insufficient water?<br>Q6. What do you personally think are the biggest barriers to sufficient household water acquisition? Why?<br>Q7. What else should we be considering with regard to household water acquisition and use (that we haven't already covered in the preceding questions)? Please include any other thoughts you may have.

**Delphi Round 2 questions**
Q1. The definitions of household water security that we received focused on 5 major aspects: characteristics of water (quality, quantity, etc.), uses for water, access to water, persons impacted by water insecurity, and time devoted to obtaining and managing household water supply (treatment, acquisition, storage, carrying, etc.). Do you agree those aspects are most essential? Are there others?<br>Q2. Here's a definition of household water security of water security based on Round 1 responses: *Safe and reliable physical, economic, and a political access to adequate quantities and quality of water for consumption (e.g. drinking and cooking), household production (e.g. crops and livestock), and cleanliness (e.g. cleaning, personal hygiene and sanitation) by all household members across all months of the year.* How would you modify and/or improve this definition?<br>Q3. In the last round we asked about the most important household activities that would be impacted by insufficient water in an open-ended way. The most frequently mentioned are those below (in no particular order). Please rank them in the order that you think to be most important in a low-resource setting:<br>1) Drinking<br>2) Cooking<br>3) Hand washing<br>4) Body washing<br>5) Laundry<br>6) Feces removal<br>7) Agriculture<br>8) Household cleaning<br>Q4**.** In the last round the modal responses to the most important household activities that were impacted by insufficient water were:<br>1) Drinking<br>2) Cooking, washing hands, bathing (tied)<br>3) Laundry, basic sanitation (tied)<br>4) Agricultural activities and household cleaning (tied)<br><br>Is there anything in this list that is striking to you? Do you disagree with the way these activities are ordered? Why or why not?<br>Q5. In the last round we asked the most severe consequences of household water insecurity in an open-ended way. The most frequently mentioned are those below (in no particular order). Please rank them in the order of consequences that you think to be most severe to least severe.<br>1) Illness/infectious diseases<br>2) Economic impacts (including those resulting from missed work)<br>3) Dehydration<br>4) Decreased agricultural production (livestock and/or crops)<br>5) Nutritional consequences/hunger<br>6) Prevention of education<br>7) Poor sanitation

8) Other________
Q6. In the last round, the modal responses to the most severe consequences of household water insecurity were:
1) Illness/Infectious diseases
2) Economic impacts
3) Dehydration
4) Decreased agricultural production
5) Nutritional consequences and hunger
6) Emotional distress
7) Prevention of education
8) Poor sanitation

Is there anything in this list that is striking to you? Do you disagree with the way these activities are ordered? Why or why not?
Q7. In the last round, we asked the biggest barriers to sufficient household water acquisition. The most frequently mentioned are those below (in no particular order). Please rank them in the order of most significant to least significant barriers:
1) Costs
2) Lack of infrastructure
3) Distance to a water source/lack of water in the household
4) Water quality
5) Ecological/environmental factors
6) Social and political structures
Q8. In the last round, the modal responses to the biggest barriers to sufficient household water acquisition were:
1) Costs (economic and labor)
2) Lack of infrastructure
3) Distance to a water source/lack of water in the household
4) Water quality
5) Ecological/environmental factors
6) Social and political structures

Is there anything in this list that is striking to you? Do you disagree with the way these activities are ordered? Why or why not?
Q9. In the last round, we asked what else we should be considering with regard to household water acquisition and use. The most common responses were:
1) Groups restricting/controlling access to serve private interests
2) Political infrastructure
3) Water-borne diseases and water harvesting
4) Coping mechanisms
5) Environmental stressors

Is there anything in this list that is striking to you? Do these trigger for you ideas about anything else we should consider?
Q10. A few respondents mentioned social dimensions of water sharing, which was really valuable because it was something we had overlooked. Please give 4-5 examples of how water acquisition and use could influence social relationships.
Q11. Currently, our Kenyan cohort is being surveyed about their experiences with water in the past 4 weeks. Do you think this is an appropriate time period for accurate recall? Why or why not?
Q12**.** Water security is currently being assessed using a metric of never (0 times), rarely (1-2 times), sometimes (3-10 times), often (>10 times) in the past 4 weeks. Do you feel like these are appropriate increments? What would you suggest instead?
Q13. Are there other thoughts on household water insecurity that you would like to share?

Delphi Round 3 questions

Q1. Here's what we think will be a final definition of household water security based on feedback we have received from previous survey rounds and our focus group discussions in the field.

Safe, sustainable and reliable physical, economic, political and social access to adequate quantities and quality of water for consumption (e.g. drinking and cooking), household production (e.g. crops and livestock), and cleanliness (e.g. ablutions, cleaning, personal hygiene and sanitation) by all household members at all times.

How would you modify and/or improve this definition?
Q2. In the last round, we asked you about the most severe consequences resulting from household water insecurity. The responses were ranked from most severe to least severe and the modal responses were:
1) Dehydration
2) Illness/infectious disease
3) Nutrition, poor sanitation
4) Decreased agricultural production (livestock and/or crops)
5) Economic impacts (including those resulting from missed work)
Q3. In the last round, we asked you the biggest barriers to household water acquisition. The responses were ranked from most concerning to least concerning barriers, and the modal responses were as follows:

1) Poor infrastructure
2) Social and political structures (government bodies, informal governance)
3) Costs (economic and labor)
4) Distance to a water source
5) Water quality
6) Ecological/environmental barriers

Would you agree with this ranking? Why or why not? Are there other barriers you find to be more critical?
Q4. In the last round, we asked you to brainstorm 4 or 5 activities about the social dimensions of water acquisition, use, and intra-household distribution. The responses were primarily classifiable into the following categories:

1) Conflict (e.g. between collectors and users, between providers and users)
2) Community dynamics (e.g. community connections around water source, water source as a place of community information dispersion, water sharing/borrowing among households, group water collection)
3) Household dynamics (e.g. strain in relationships when women spend time gathering water, distribution of water among household members, resentment within household due to gendered nature of household water acquisition)
4) Education (e.g. community members teaching each other how to treat water and wash hands, children missing school to gather water)
5) Gender (e.g. reinforcement or challenge of gender roles with regard to water acquisition, household division of labor)

Do you agree with these social aspects of water use? Are there other social dimensions of water that we haven't previously considered?
Q5. The religious and spiritual aspects of water is one domain that we feel may be under-explored in our work. Can you think of any domains of water acquisition and use related to religion or spirituality?
Q6. In our review of the literature, we are finding that there may be social unacceptable ways in which people access and/or acquire water. Please list 3 or 4 coping strategies you've seen or can imagine in water insecure households.
Q7. Currently, we plan to administer the household water insecurity scale several times within the same household across 1 year. Responses to the frequency of experiences of water insecurity include categories of times encompassing never, rarely, sometimes, and often within the last month. In your own opinion, how many days is "rarely" in the last month? "Sometimes?" "Often?"
Q8. Is there anything else we should be thinking of as we develop a household-level water insecurity scale?
