# Supplementary material for: A novel household water insecurity scale: Procedures and psychometric analysis among postpartum women in western Kenya
Source: PLoS One. 2018 Jun 8;13(6):e0198591. doi: 10.1371/journal.pone.0198591 (PMC5993289; doi:10.1371/journal.pone.0198591)
Supplement: S2 Table — (PDF) [file pone.0198591.s005.pdf]

S2 Table. Initial and final household water insecurity scale items for use among postpartum women in Kenya

Final items	HHWI Scale Items
X	Q1) In the last 4 weeks, how frequently did you **WORRY** you would not have **enough water for all** of your household needs? *
	Q2) In the last 4 weeks, how frequently have you **lacked money needed** to buy water?*
X	Q3) In the last 4 weeks, how frequently did you feel **angry or frustrated** that you would not have **enough water for all** of your household needs? *
	Q4) In the last 4 weeks, how frequently have you **thought of leaving (name of town)** because there was no water there?
X	Q5) In the last 4 weeks, how frequently have you **worried about the safety** of the person getting water for your household? By getting, I mean: traveling to, drawing the water, and returning with the water.
	Q6) In the last 4 weeks, how frequently has the household **not had enough** water for your **garden/shamba or crops?**
	Q7) In the last 4 weeks, how frequently has there **not been enough water** to give to your **animals and poultry**?
X	Q8) In the last 4 weeks, how frequently has the time **spent fetching** water prevented anyone in your household from **earning money**?*
X	Q9) In the last 4 weeks, how frequently has the **time spent fetching** water prevented you or anyone in your household from **caring for your children**?
	Q10) In the last 4 weeks, how frequently has the **time spent fetching** water prevented anyone in your household from **doing household chores** (such as cooking, preparing food, washing clothes, etc.)?*
	Q11) In the last 4 weeks, how frequently did the **children** in your household **miss school** because they were **collecting water**?
X	Q12) In the last 4 weeks, how frequently has anyone in your household asked to **borrow water** from other people?
	Q13) In the last 4 weeks, how frequently have you **loaned water** to anyone?
X	Q14) In the last 4 weeks, how frequently has there **not been enough water** in the household to **wash clothes**?*
X	Q15) In the last 4 weeks, how frequently have you **missed meetings** in your community (church, funerals, community meetings, etc.) because there wasn't enough water?*
X	Q16) In the last 4 weeks, how frequently have you missed meetings in your community (church, funerals, community meetings, etc.) because you lacked water to take a bath and you **felt too dirty to go**?
X	Q17) In the last 4 weeks, how frequently have you or anyone in your household had to **change what was being cooked** because there wasn't enough water?*
X	Q18) In the last 4 weeks, how frequently have you or anyone in your household had to go **without washing hands** after defecating, changing diapers or other **dirty** activities because you didn't have enough water?*
X	Q19) In the last 4 weeks, how frequently have you not had enough water to **wash your children's face and hands**?
X	Q20) In the last 4 weeks, how frequently have you or anyone in your household had to go without **washing their body** because there wasn't enough water?*
X	Q21) In the last 4 weeks, how frequently did you want to **treat your water**, but couldn't? By treat, I mean boiling, using chemicals to treat, or other ways you make your water safe to use or drink.
X	Q22) In the last 4 weeks, how frequently have you or anyone in your household **actually** had to **drink water** that you thought was unsafe?
X	Q23) In the last 4 weeks, how frequently did you have problems with water that caused **arguments/trouble** with **neighbors** or others in the community? *
	Q24) In the last 4 weeks, how frequently did you have problems with water that caused **arguments/trouble** with people **within your household**?
X	Q25) In the last 4 weeks, how frequently has there **not been as much water to drink as you would like** for you or members of your household?

**X**	Q26) In the last 4 weeks, how frequently have you or anyone in your household not had **enough water** to **take medications**?
	Q27) In the last 4 weeks, how frequently have you **not collected water where you wanted to** because you were **too sick or weak** to collect water?*
**X**	Q28) In the last 4 weeks, how frequently have you or anyone in your household gone to **sleep thirsty**?*
**X**	Q29) In the last 4 weeks, how frequently have you had **no water whatsoever** in your household?

Notes: Items with asterisks (*) represent those items drawn from existing studies in developing the initial set of household water insecurity scale questions and those without the asterisks are items that were added from our formative work (activities A & B)
