# Supplementary material for: A novel household water insecurity scale: Procedures and psychometric analysis among postpartum women in western Kenya
Source: PLoS One. 2018 Jun 8;13(6):e0198591. doi: 10.1371/journal.pone.0198591 (PMC5993289; doi:10.1371/journal.pone.0198591)
Supplement: S3 Table — (PDF) [file pone.0198591.s006.pdf]

S3 Table. Description of model fit indices and thresholds used in evaluating scale development results

**Model Fit Indices**	**Description**	**Thresholds used in this study**	**References**
Chi-square test	The chi-square value is a test statistic of the goodness of fit of a factor model. It compares the observed covariance matrix with a theoretically proposed covariance matrix.	Chi-square test of model fit has been assessed to be overly sensitive to sample size and to vary when dealing with non-normal variables. Hence, the use of a non-normal data, a small sample size (180-300) and highly correlated items make the chi-square approximation inaccurate. We do not refer to this index for model fitness in this study.	[74, 61]
Root Mean Square of Error of Approximation (RMSEA)	RMSEA estimates "the difference between the examined model and a hypothetical model where every component in the model is related to every other component" [75:3]. The RMSEA is considered as a "badness-of-fit index where a value of zero indicates the best fit" [74:205].	Browne and Cudeck have suggested that RMSEA≤0.05 may indicate a good fit. Range should be 0≤RMSEA≤0.10.	[74,75,79,80]
Tucker Lewis Index (TLI)	TLI is based on the idea of comparing the proposed factor model to a model in which no interrelationships at all are assumed among any of the items.	Bentler and Bonnett suggest that models with overall fit indices of less than 0.90 are generally inadequate and can be improved substantially. Hu and Bentler recommend TLI≥0.95. This study used TFI≥0.95.	[76, 77]
Comparative Fit Index (CFI)	CFI is an incremental relative fit index that "measures the relative improvement in the fit of [a] researcher's model over that of a baseline model".	This study used CFI≥0.95 for 'an acceptable fit'	[74,76]
Standardized Root Mean Square Residual (SRMR)	SRMR is a measure of the mean absolute correlation residual, the overall difference between the observed and predicted correlations.	Threshold for acceptable model fit is SRMR≤0.08.	[71,74,76,78]

S3 Table. Description of model fit indices and thresholds used in evaluating scale development results

**Model Fit Indices**	**Description**	**Thresholds used in this study**	**References**
Weighted Root Mean Square Residual (WRMR)	WRMR uses "a variance-weighted approach specially suited for models whose variables measured on different scales or have widely unequal variances"; It has been assessed to be most suitable in assessing models from non-normal data.	Threshold for assessing WRMR should be <1.0.This index is used only for confirmatory factor analysis.	[61,75]
Standard of Reliability for scales	Nunnally suggests that in making important decisions on scales a reliability of 0.90 is the minimum threshold that should be tolerated while a reliability of 0.95 should be the desirable standard	Threshold for assessing internal consistency for scales should be **≥ 0.90**	[92,101]
